# Critical Events in Anaesthetised Kids Undergoing Tracheal Intubation (CRICKET)—study protocol for an international multicentre prospective observational study

**DOI:** 10.1016/j.bjao.2025.100523

**Published:** 2026-02-07

**Authors:** Tom Bennett, Nicola Disma, Thomas Engelhardt, John Fiadjoe, Alexander Fuchs, Annery Garcia-Marcinkiewicz, Dario Gregori, Robert Greif, Walid Habre, Giulia Lorenzoni, Maren Kleine-Brueggeney, Clyde Matava, Cinzia Anna Maria Papappicco, Vinicius Caldeira Quintão, Thomas Riva, Britta S. von Ungern-Sternberg, Eugene Zoumenou

**Affiliations:** 1 Department of Anaesthetics, University Hospital Southampton, Southampton, UK; 2 Unit for Research in Anesthesia, IRCCS Istituto Giannina Gaslini, Genoa, Italy; 3 Department of Pediatric Anesthesia, Montreal Children's Hospital, Montreal QC, Canada; 4 Department of Anesthesiology and Critical Care Medicine, Children's Hospital of Philadelphia, Philadelphia, PA, USA; 5 Department of Anaesthesiology and Pain Medicine, Inselspital, Bern University Hospital, University of Bern, Bern, Switzerland; 6 Unit of Biostatistics, Epidemiology and Public Health, Department of Cardiac, Thoracic, Vascular Sciences and Public Health, University of Padova, Padua, Italy; 7 University of Bern, Bern, Switzerland; 8 Department of Cardiac Anesthesiology and Intensive Care Medicine, Deutsches Herzzentrum der Charité (DHZC)—Medical Heart Center of Charité and German Heart Institute Berlin, Berlin, Germany; 9 Department of Anesthesiology, Critical Care and Pain Medicine, Boston Children's Hospital, Boston, MA, USA; 10 Charité–Universitätsmedizin Berlin, corporate member of Freie Universität Berlin and Humboldt–Universität zu Berlin, Berlin, Germany; 11 German Center for Cardiovascular Research (DZHK), partner site Berlin, Berlin, Germany; 12 Department of Anesthesia & Pain Medicine, The Hospital for Sick Children, Toronto, ON, Canada; 13 Department of Anesthesiology and Pain Medicine, University of Toronto, Toronto, ON, Canada; 14 Department of Pediatrics, Hospital das Clínicas HCFMUSP, Faculdade de Medicina, Universidade de São Paulo, São Paulo, Brazil; 15 Instituto da Criança e do Adolescente ICr, Hospital das Clínicas HCFMUSP, Faculdade de Medicina, Universidade de São Paulo, São Paulo, Brazil; 16 Department of Anaesthesia and Pain Medicine, Perth Children's Hospital, Child and Adolescent Health Service, Nedlands, WA, Australia; 17 Institute for Paediatric Perioperative Excellence, The University of Western Australia, Perth, WA, Australia; 18 Division of Emergency Medicine, Anaesthesia and Pain Medicine, The University of Western Australia, Perth, WA, Australia; 19 Perioperative Care Program, Perioperative Medicine Team, The Kids Research Institute Australia, Nedlands, WA, Australia; 20 Hubert Koutoukou Maga National University Hospital, Cotonou, Benin

**Keywords:** airway management, anaesthesia critical events, paediatric anaesthesia, tracheal intubation

## Abstract

**Background:**

Critical Events in Anaesthetised Kids undergoing Tracheal Intubation (CRICKET) is a prospective, international multicentre observational study with the objective of capturing, assessing, and analysing critical events associated with tracheal intubation in children.

**Methods:**

CRICKET involves paediatric patients aged 0–16 yr, requiring tracheal intubation, performed by the anaesthesia team for procedures or interventions requiring general anaesthesia, either planned or unplanned. Patient characteristics and airway management techniques are recorded using a dedicated electronic case report form. If one or more critical events associated with airway management occur, a second, more detailed questionnaire will be completed for the follow-up until the patient is discharged or up to a maximum of 30 days. We aim to include 105 000 patients from participating centres disseminated worldwide. Every participating centre collects data over a consecutive observational period of 3 months. The primary outcome is the incidence of critical events associated with tracheal intubation in children.

**Results:**

The CRICKET study started in January 2024 and is currently ongoing. By May 2025, around 25 000 patients were entered into the database, with an estimated 50 000 patients by the end of 2025. Because of the observational nature of the study and the extensive international involvement and effort, continuing the study or analysing the existing data will depend on available resources and exact incidence of critical events.

**Funding:**

This work was supported by the Italian Ministry of Health (Ricerca Corrente 2025).

**Clinical trial registration:**

ClinicalTrials.gov (NCT 05804188).

Tracheal intubation-associated events (TIAEs) can lead to immediate life-threatening complications and long-term sequelae, especially for very young patients and those affected by comorbidities.^1^^,^^2^ The fourth National Audit Project (NAP4)^3^ in the UK estimated that major complications during airway management occur with an incidence of up to 1 in 5500 anaesthesia cases in adults and children. In this audit, the few reported cases with a ‘cannot intubate cannot oxygenate’ (CICO) situation had very severe consequences (death, brain damage, prolonged hospitalisation, unplanned ICU admission). The APRICOT study^4^ reported an incidence of difficult airway, defined as Cormack–Lehane (CL) grading of 3 or 4 or three or more attempts to insert the tracheal tube, of 0.9%, increasing in neonates to 5%, with 40% of these patients suffering from hypoxemia and 8% from hypoxemia and bradycardia. Fortunately, only a small number of severe TIAEs led to long-term morbidity or increased mortality. This low rate of serious sequelae mirrors findings from NECTARINE^5^ and PeDI,^1^^,^^6^ and likely reflects the benefits of early recognition and timely intervention.

Infants and children experience a higher rate of airway-related critical events owing to age-dependent anatomical and physiological features, and syndromic conditions and comorbidities. Therefore, they deteriorate rapidly if not expertly managed. Human factors and variable team performance further influence outcomes, underscoring why current guidelines^2^^,^^7^^,^^8^ advocate limiting attempts, seeking help early, and structured debriefing. Despite these recommendations, practice remains heterogeneous. The Critical Events in Anaesthetised Kids undergoing Tracheal Intubation (CRICKET) study is therefore crucial, providing contemporary, real-world data on paediatric airway management, identifying where gaps persist, and defining priorities to enhance safety and reduce preventable harm.

Where earlier studies have reported critical events in broad terms, often limited to severe outcomes such as cardiac arrest or death, the CRICKET study will address this gap by prospectively capturing detailed, standardised data on a vast number of airway-related critical events during tracheal intubation in children up to 16 yr of age. The inclusion of centres with diverse geographic and economic backgrounds enhances the generalisability and external validity of the study's findings.

## Methods

The primary aim is to determine the incidence of paediatric patients presenting with critical events associated with tracheal intubation. Table 1 presents definitions of critical events used in this study. As an observational study, CRICKET remains inherently susceptible to confounding by indication, although its large sample size and detailed, standardised data collection will help to characterise and mitigate this potential bias. Analysis of these events will focus on identifying clusters of events and risk factors for frequently occurring events. For example, in some conditions, critical events might be characterised by two or more airway-related complications occurring in close temporal proximity during the same intubation episode, suggesting a connected cascade (such as desaturation followed by bradycardia and hypotension) rather than isolated, unrelated events. Similarly, clusters of particular events might be identified in a specific subgroup of patients. This may allow us to identify independent risk factors and, consequently, children at particular risk for critical events.

**Table 1:** Definition of critical events associated with tracheal intubation. CPAP, continuous positive airway pressure.

Definition of tracheal intubation-associated critical events	
‘Critical events’ in this study and time frame	Any episode of occurrence during tracheal intubation requiring a medical intervention from the start of anaesthesia until the end of anaesthesia (defined as handover to either the PACU, the paediatric or neonatal intensive care unit, the ward, or discharge home straight from anaesthesia care).
Severe hypoxemia	SpO_2_ <85% or >20 points below initial value for at least 60 s.
Severe bradycardia	Persistent bradycardia for at least 1 min: • 0–3 months old: HR <80 beats min^−1^ • 4 months–2 yr: HR <60 beats min^−1^ • 2–10 yr old: HR <40 beats min^−1^ • 10–16 yr old: HR <30 beats min^−1^
Oesophageal intubation	Tracheal tube placed in the oesophagus diagnosed by (video-) laryngoscopy, absence of sustained EtCO_2_ trace, absence of lung ventilation (auscultation or absence of chest excursions) causing a decrease in oxygenation.
Laryngospasm	Complete airway obstruction associated with rigidity of the abdominal and chest walls and leading to unsuccessful child’s ventilation, or glottic closure associated with chest movement but silent unsuccessful child’s respiratory efforts and assisted ventilation, unrelieved in both situations with simple jaw thrust and CPAP manoeuvres and requiring the administration of medication (propofol, suxamethonium etc.), tracheal (re)-intubation, or both.
Bronchospasm	Increased respiratory effort, especially during expiration, and wheeze on auscultation. Episode of bronchospasm requires the administration of a bronchodilator.
Stridor after extubation	Severe inspiratory flow limitation with sternal retraction, intrathoracic pressure swing, and potentially cyanosis occurring after extubation with or without the administration of oxygen; i.v. steroids, epinephrine (nebulisation), or both; or tracheal intubation. This can be documented clinically or with diagnostic examination, with persistence of symptoms.
Obstruction of tracheal tube	Obstruction of tracheal tube needing lavage or tube exchange.
Airway bleeding	Acute bleeding from nose, arytenoids, or pharynx causing obstruction or risk for pulmonary aspiration.
Can’t intubate can’t oxygenate (CICO)	Situation when there is failed intubation and failure to adequately oxygenate using facemask ventilation or supraglottic airway device resulting in increasing hypoxemia in an anaesthetised and paralyses patient.
Severe bradycardia/cardiac arrest	Cessation of circulation (no pulse) or severe bradycardia requiring chest compressions, during the intubation/extubation manoeuvres.
Pulmonary aspiration	Presence of non-respiratory secretions (gastric, particulate, blood) in the airway as evidenced by (video-)laryngoscopy, suctioning, or bronchoscopy or radiologic signs.
Pneumothorax/pneumomediastinum	Air in the thorax, mediastinum, or both as a consequence of tracheal intubation and ventilation, causing lung collapse or mediastinum dislodgment diagnosed by lung ultrasound, X-ray, or both.
Negative pulmonary oedema	Non-cardiogenic pulmonary oedema that results from the generation of high negative intrathoracic pressure needed to overcome upper airway obstruction.

In accordance with Pediatric Advanced Life Support (PALS) guidance,^9^ bradycardia will be defined as a sustained age-specific slow heart rate with clinical compromise and differentiated from tachyarrhythmias or rhythm disturbances that may also cause haemodynamic instability, but require substantially different treatments. Tachyarrhythmias will be recorded separately, if they occur, as critical events during airway management.

### Study design

CRICKET is a prospective, international, multicentre observational study collecting health-related data over a consecutive 3-month period. Centre recruitment will be sequential between January 2024 and December 2025, with each site initiating its observation window upon ethics approval and confirmation of adequate local resources. During the study period, designated research staff or the anaesthetist in charge of the patient will complete a dedicated case report form (CRF) for every child undergoing general anaesthesia with tracheal intubation, whether elective or unplanned. Importantly, CRF data are collected exclusively for research purposes and are entirely separate from the medical record. For cases without critical events, data collection ends at anaesthesia completion and tracheal extubation. When one or more critical events occur, an additional detailed section of the CRF and a structured 24-h follow-up will be completed to document management and possible sequelae. To minimise reporting bias and alleviate concerns about disclosure, data are recorded anonymously, no identifiable staff information is collected, participating centres are not benchmarked against each other, and the study explicitly adopts a quality-improvement-oriented framework to encourage accurate and complete reporting. Any missing data will be actively queried by the local study team to ensure completeness, and variables that remain unresolved after these efforts will be handled using predefined statistical procedures appropriate for observational datasets, including sensitivity analyses and multiple imputations to assess the impact of missingness.

**Fig 1:**
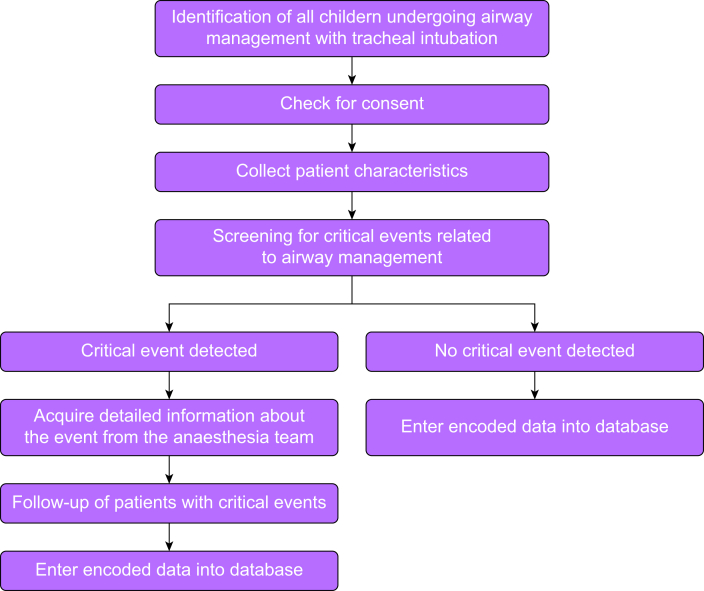
Study flowchart.

The collected health-related data will be entered into an electronic research database in REDCap® (Vanderbilt University Medical Center, 2215 Garland Ave, Nashville, Tennessee, USA) (Research Electronic Data Capture)^10^, ^11^, ^12^, ^13^, ^14^, ^15^ (see Data Management section). In this database, data will be encoded to ensure that neither patients nor providers are identifiable. The study flowchart is presented in Figure 1.

### Outcome measurements

#### Primary outcomes

The primary study outcome is the incidence of anaesthesia cases with critical events (definitions in Table 1) associated with airway management requiring tracheal intubation, from the start of anaesthesia until the discharge of the patient from the PACU or end of anaesthesia (defined as handover to the paediatric or neonatal intensive care unit, the ward, or discharge home straight from anaesthesia care) in children aged 0–16 yr.

#### Secondary outcomes

Secondary study outcome parameters are the incidences of the critical events associated with tracheal intubation and the number of critical events per case, if multiple occurred during the same case. Risk factors of critical events will also be identified.

Children with critical events associated with tracheal intubation will be followed up until complete resolution of the event or for a maximum of 30 days whichever comes earlier.

### Study population and eligibility criteria

Patients are eligible if they fit the following inclusion criteria:

• All paediatric patients, aged 0–16 yr, requiring tracheal intubation, either planned or unplanned, performed by the anaesthesia team for procedures or interventions requiring general anaesthesia.
• Cases in which a surgeon performs the intubation, whether electively or as a rescue, will be included. Likewise, patients who already have an airway in place (e.g. tracheostomy or tracheal tube) and subsequently undergo airway removal followed by a new intubation will also be captured.

Exclusion criteria are as follows:

• Patients >16 yr.
• Patients admitted to the operating room already intubated and not requiring a tube exchange.
• Refusal to give consent or withdrawal of consent if such is required by the relevant ethics committee.

### Study sites and recruitment procedures

We aim to recruit both academic and non-academic anaesthesia departments worldwide, including dedicated children’s hospitals and centres with mixed paediatric and adult practice across all continents, in order to capture a broad range of paediatric airway management approaches. The study was announced at international anaesthesia meetings and through a dedicated website (https://cricketstudy.eu/), with an open call for participating centres. Each participating institution is required to submit the protocol for approval to its local ethics committee.

Given the multinational nature of the study, requirements for informed consent will follow local ethics committee determinations, including the use of waivers where permissible. To address the potential for selection bias introduced by differing consent pathways, a planned sensitivity analysis will compare centres using prospective individual consent with those operating under a waiver or alternative approval model.

## Results

The CRICKET study is currently ongoing. In May 2025, around 25 000 patients were entered into the database, with a projected 50 000 patients by the end of 2025. Due to the observational nature of the study and the extensive international involvement and effort, the steering committee decided to stop recruiting at the end of 2025 and proceed with database cleaning, subsequent statistical analysis, and publication.

## Discussion

### Assessments of outcomes for data quality

For every patient, the anaesthesia provider will complete an electronic questionnaire in REDCap®, together with an expanded form whenever a critical event related to tracheal intubation occurs. Because complications may arise at any time during anaesthesia, treating clinicians act as an extension of the study team and are responsible for real-time, case-specific data entry; however, these study forms remain entirely separate from the clinical record. To ensure completeness and minimise reporting bias, dedicated research staff, when available and not involved in clinical care, will perform daily checks of all cases from the preceding 24 h, reviewing submitted questionnaires for clarity and retrieving missing information. Where clarification is needed, queries may be returned to the individual clinician who performed the case, ideally close to end of the case and without collecting any identifiable information. Study personnel will not infer the occurrence of a critical event solely from the medical record: an event must be affirmatively documented by the treating anaesthesia provider. Conversely, the independent Clinical Event Committee (CEC) will adjudicate all reported events, ensuring consistency with study definitions and assigning severity levels; however, the CEC cannot overrule a clinician’s explicit confirmation that an event occurred, although it may reclassify the event type or severity based on the protocol definitions. Data quality verification will take place only after all ancillary datasets are merged into the main REDCap® database. Once all outstanding queries are resolved and data quality procedures completed, the database will be locked for statistical analysis.

### Causal model for identifying determinants of critical events

Recent cross-sectional studies have suggested a relationship between the risk of critical events in paediatric tracheal intubation and patient anthropometric characteristics, anatomical variations in the airway, the presence of comorbidities, and the skill and experience of the healthcare provider. Despite these findings, no study has investigated the causal relationship between these events and the factors underlying their occurrence. Identifying and understanding the determinants of critical events during paediatric tracheal intubation are crucial for recognising and managing them effectively, thereby enhancing the quality and safety of tracheal intubation and improving the outcomes of young patients.

To estimate causal effects, we must appropriately identify, select, and include all relevant confounding variables.^10^ We will construct a directed acyclic graph (DAG) to visually represent the causal structure of our research question.^11^ A DAG provides a non-parametric depiction of cause–effect relationships and enables us to identify variables that may plausibly confound the association between exposure and outcome.^12^^,^^13^ This approach offers an intuitive visual framework grounded in rigorous causal inference methodology.^14^^,^^15^ In the CRICKET observational study, we will develop a causal model to identify factors associated with critical events during paediatric tracheal intubation. We will use a DAG to identify potential confounders that influence the relationship between critical events and their determinants. We will draw on expert opinion to define known causal pathways and exposures. We will also perform sensitivity analyses to evaluate the influence of unmeasured confounding on our findings. We will construct the DAG using the R package [CRAN (open source), Radboud University & Medical BioSciences, Nijmegen, The Netherlands (Textor)] *dagitty*,^15^ based on the web-based DAGitty platform. To minimise bias in interpreting the results, we will develop the causal model and identify the exposures of interest in parallel with the principal statistical analysis, once recruitment is complete and data cleaning has been finalised.

### Sample size calculation

Previous studies report that respiratory critical events during paediatric airway management occur with an incidence ranging from 0.28% to 6%.^4^ We calculated the sample size using the Wilson method. To adopt a conservative approach, we assumed an expected event rate between 1% and 0.5% for the less represented age groups and explored several precision scenarios, with the half-width of the 95% confidence interval ranging from 7% to 9.5% of the estimated incidence. Selected combinations of incidence and precision, along with the corresponding sample sizes, are shown in Table 2. Specifically, our target was to obtain a 95% confidence interval of 0.9–1.1% around an expected incidence of 1%. To further ensure robustness, we calculated a conservative scenario based on a 0.5% incidence and 8.5% precision, resulting in a required sample size of approximately 105 000 patients. The number of participating centres will depend on each centre’s expected caseload during the 3-month observational window. If the required sample size is not achieved within the planned 24-month recruitment period, the steering committee may either extend recruitment or conclude enrolment and analyse the accumulated data, depending on the number of recorded events and the remaining available resources. All sample size computations were performed using the R package binomSamSize.^15^

**Table 2:** Sample size corresponding to the different scenarios of precision, considering the combinations with an event rate of 1% or 0.5% and a precision level ranging from 7% to 9.5% of the expected event rate.

Precision level	Sample size	
	1% event rate	0.5% event rate
7% of the event rate	77 702	156 099
7.5% of the event rate	67 650	136 044
8.5% of the event rate	52 714	105 897
9.5% of the event rate	42 215	84 845

### Statistical analysis

A descriptive analysis of the data will be carried out, using counts and percentages to summarise categorical data and median (interquartile range) to summarise continuous data.

The incidence of critical events will be estimated and reported together with a 95% confidence interval. Furthermore, critical event incidence will be provided for different age categories, operator’s experience, operator’s speciality, and centre volume.

In terms of risk factor analysis, a univariable and multivariable mixed-effect logistic regression model will be computed based on possible predictors, which include for example age (≤12 months), weight (≤10 kg), number of attempts (more than two), presence of comorbidities (ASA score ≥3), comorbidity related to airway management (e.g. relevant syndromes), expected difficult intubation, type of anaesthesia induction (i.v. or inhalation), no neuromuscular blocking agent given, airway surgery or ears, nose, throat (ENT) surgery, and experience level of the person performing tracheal intubation. Appendix 1 lists the relevant factors used for primary and secondary analyses. Goodness-of-fit, calibration, discriminatory capacity, and predictive skill of the regression models will be assessed using Nagelkerke pseudo-*R*^2^, calibration belts, area under the receiver operating characteristic curve (AUROC), and Brier Score, respectively.

The target sample size for CRICKET is informed by statistical considerations and the practical constraints of conducting a large, multinational observational study. Although the sample size calculation indicates that approximately 105 000 cases are required to estimate an event rate of 0.5–1% with acceptable precision, the achievable enrolment also depends on centre capacity, case volume, and the coordinated activation of participating sites across multiple jurisdictions. Recruitment will therefore proceed sequentially from January 2024 to December 2025, with each centre contributing a consecutive 3-month sampling window once local approvals and resources are in place. If the required sample size is not reached by the end of 2025, the steering committee may extend the recruitment period or, alternatively, close enrolment and proceed with analysis of the accrued data, depending on the number of events captured and the resources remaining.

Missing data will be characterised according to recognised mechanisms: missing completely at random (MCAR), where the probability of missingness is unrelated to patient characteristics or outcomes; missing at random (MAR), where missingness depends on observed data; and missing not at random (MNAR), where missingness is related to unobserved factors. We will first explore patterns of missingness to determine the most plausible mechanism. For MCAR or MAR data, we plan to apply appropriate imputation strategies, such as multiple imputation using chained equations, to minimise bias and preserve statistical power. If data are suspected to be MNAR, we will conduct sensitivity analyses to assess the robustness of our findings under varying assumptions about missing values. These procedures will follow best practices for handling missing data in large-scale observational studies. If no imputation is decided, analyses will be performed within the framework of a complete case analysis.

### Data management

Patient characteristics will be obtained from the anaesthesia record. All study data will be directly recorded in the CRF on paper or electronically, which are considered as source data. Dedicated study personnel will check all data entry into the CRFs for their validity and completeness on a weekly basis. External monitoring will not occur, as this is an investigator-initiated multicentre study and quality measures must be implemented at each study centre.

Study data generated in participating centres will be collected and managed using a web application on the servers of the University of Padova using the study management software REDCap®, set up on a dedicated website with all the guarantees of security requirements. Study data will be collected and managed using REDCap® electronic data capture tools, developed, tested, validated, and hosted at the Department of Cardiac-Thoracic-Vascular Sciences and Public Health, University of Padova, Italy. REDCap® is a secure, web-based software platform designed to support data capture for research studies, providing (1) an intuitive interface for validated data capture; (2) audit trails for tracking data manipulation and export procedures; (3) automated export procedures for seamless data downloads to common statistical packages; and (4) procedures for data integration and interoperability with external sources. Access to REDCap® will be granted only to data collecting staff of participating centres, in accordance with the procedures outlined in the protocol. Each participating centre is granted access only to the patient data it has generated and recorded on the REDCap® platform. The data will be recorded using an encrypted data connection (HTTPS) in input masks via a web browser or mobile app.

Study data will be archived for a minimum of 10 yr after study termination or premature termination of the clinical trial.

### Ethics

The CRICKET study has been approved by Swiss Ethics Committee (approval no. 2023-00246 on 9 March 2023). Any amendment to the protocol must be approved by the relevant ethics committees/authorities. The study was prospectively registered at www.clinicaltrials.org (identifier: NCT 05804188).

Study centres will enter data in the centralised database and will have access only to their own institutional data. The centralised database will be established in Padua, Italy, and will be subject to the data protection laws in force in Europe (General Data Protection Regulation, 2016/679). Access to the data will only be granted for data control and statistical analysis by the person responsible for the centralised database. By these measures, we intend to achieve maximal protection of patient and institutional data.

### Publication and dissemination policy

The results will be published in a peer-reviewed scientific journal to provide new knowledge that may support the development of targeted quality improvement strategy in paediatric airway management, including modifications to clinical strategies, enhanced training, or addressing technical gaps. Final decision on publishing the results will be kept by the steering committee of the study. Authors of the publication will be team members of the consortium who contributed to the design, conduct, or analysis of the study and who approved the final version of the manuscript. International, national, and local investigators will be listed as collaborators (‘CRICKET Investigator Group’) in all publications that include CRICKET data, including secondary and ancillary analyses for subsequent publications.

## Patient and public involvement

Patients and the public were not involved in the design, conduct, or reporting, or dissemination plans of this research.

## Authors’ contributions

Concepted the protocol and designed the CRF: all authors

Designed the statistical analysis plan: DG, GL

Designed the electronic CRF: DG, GL, CAMP

Drafted the article: ND, TR, CAMP

Equally contributed to developing and approving the protocol and reading and approving the present version of the paper: all authors

## Funding

Research fund of the Department of Anaesthesiology and Pain Medicine, Inselspital, Bern, Switzerland; the Unit for Research of IRCCS Istituto Giannina Gaslini, Genova, Italy; The cost of open access was supported by the Italian Ministry of Health (Ricerca Corrente 2025); Stiftung für die Forschung in Anästhesiologie und Intensivmedizin (Bern, Switzerland); European Society of Anaesthesiology and Intensive Care through the Paediatric Anaesthesia Research Network Research Group (https://esaic.org/research/research-groups/parnet/); Stan Perron Charitable Foundation (Perth, Australia); National Health and Medical Research Council, Australia; Smile Train, Inc., New York, NY, USA (grant 0206904).

## Declaration of interest

The authors declare that they have no conflicts of interest.

## References

[1] Fiadjoe J.E., Nishisaki A., Jagannathan N. (2016). Airway management complications in children with difficult tracheal intubation from the Paediatric Difficult Intubation (PeDI) registry: a prospective cohort analysis. Lancet Respir Med.

[2] Apfelbaum J.L., Hagberg C.A., Connis R.T. (2022). 2022 American Society of Anesthesiologists practice guidelines for management of the difficult airway. Anesthesiology.

[3] Cook T.M., Woodall N., Harper J., Benger J. (2011). Major complications of airway management in the UK: results of the Fourth National Audit Project of the Royal College of Anaesthetists and the Difficult Airway Society. Part 2: intensive care and emergency departments. Br J Anaesth.

[4] Habre W., Disma N., Virag K. (2017). Incidence of severe critical events in paediatric anaesthesia (APRICOT): a prospective multicentre observational study in 261 hospitals in Europe. Lancet Respir Med.

[5] Disma N., Virag K., Riva T. (2021). Difficult tracheal intubation in neonates and infants. NEonate and Children audiT of Anaesthesia pRactice IN Europe (NECTARINE): a prospective European multicentre observational study. Br J Anaesth.

[6] Stein M.L., Sarmiento Argüello L.A. (2024). Airway management in the paediatric difficult intubation registry: a propensity score matched analysis of outcomes over time. EClinicalMedicine.

[7] Disma N., Asai T., Cools E. (2024). Airway management in neonates and infants: European Society of Anaesthesiology and Intensive Care and British Journal of Anaesthesia joint guidelines. Br J Anaesth.

[8] Disma N., Asai T., Cools E. (2024). Airway management in neonates and infants: European Society of Anaesthesiology and Intensive Care and British Journal of Anaesthesia joint guidelines. Eur J Anaesthesiol.

[9] Lasa J.J., Dhillon G.S., Duff J.P. (2025). Part 8: Pediatric Advanced Life Support: 2025 American Heart Association and American Academy of Pediatrics guidelines for cardiopulmonary resuscitation and emergency cardiovascular care. Circulation.

[10] Krieger N., Davey Smith G. (2016). The tale wagged by the DAG: broadening the scope of causal inference and explanation for epidemiology. Int J Epidemiol.

[11] Greenland S., Brumback B. (2002). An overview of relations among causal modelling methods. Int J Epidemiol.

[12] Greenland S., Pearl J., Robins J.M. (1999). Causal diagrams for epidemiologic research. Epidemiology.

[13] Jenkins L., Chang W., Buscemi V. (2019). Is there a causal relationship between acute stage sensorimotor cortex activity and the development of chronic low back pain? A protocol and statistical analysis plan. BMJ Open.

[14] Shrier I., Platt R.W. (2008). Reducing bias through directed acyclic graphs. BMC Med Res Methodol.

[15] Ankan A., Wortel I.M.N., Textor J. (2021). Testing graphical causal models using the R package “dagitty”. Curr Protoc.

