# Supplementary material for: Critical Events in Anaesthetised Kids Undergoing Tracheal Intubation (CRICKET)—study protocol for an international multicentre prospective observational study
Source: BJA Open. 2026 Feb 7;17:100523. doi: 10.1016/j.bjao.2025.100523 (PMC12907495; doi:10.1016/j.bjao.2025.100523)
Supplement: Multimedia component [file mmc1.docx]

**Appendix 1**. Factors to be used for primary and secondary analysis.

***Primary analysis***:

- Age groups, specifically prematurity, neonatal age and < 1 year of age
- Cranio-facial abnormality
- Congenital abnormality syndrome
- Current upper respiratory tract infection (e.g. Laryngitis/croup)
- Degree of urgency (elective vs emergency indication)
- Type of procedure (surgery vs procedure vs imaging)
- **American Society of Anaesthesiologists Physical Status Classification System (**ASA) III-IV and Paediatric Risk Assessment Model (PRAM)
- Anticipated difficult intubation (e.g. History of previous difficult intubation)
- Level of airway operator experience
- Specialty of the operator performing tracheal intubation
- Cormack & Lehane / Percentage of glottic opening (POGO) score

***For secondary analysis***:

- Type of procedure (ENT, craniofacial, thoracic)
- Type of anaesthesia
- Technique of induction
- Neuromuscular blocking agent (NMBA)
- Nasal vs oral intubation
- Preoxygenation
- Type of device of intubation
- Supplemental oxygen during the attempt
- Known lung pathology or cardiac pathology
- Asthma/bronchiolitis
- Location of intubation different from Operating Room
